# Synthesis of Vanadium Carbide by Mechanical Activation Assisted Carbothermic Reduction

**DOI:** 10.3390/ma13194408

**Published:** 2020-10-02

**Authors:** Zaki I. Zaki, Mohamed H. El-Sadek, Heba H. Ali, Hesham Ahmed

**Affiliations:** 1 Department of Chemistry, College of Science, Taif University, P.O. Box 11099, Taif 21944, Saudi Arabia; zakimohamed@tu.edu.sa; 2 Pyrometallurgy Department, Minerals Technology Division, Central Metallurgical R&D Institute (CMRDI), P.O. Box 87, Cairo 11865, Egypt; hebahma@cmrdi.sci.eg; 3 Department of Civil, Environmental and Natural Resources Engineering, Minerals and Metallurgical Engineering, MiMeR, Luleå University of Technology, 97187 Luleå, Sweden

**Keywords:** mechanical activation, carbothermic reduction, vanadium oxide, carbon black, vanadium carbide

## Abstract

Vanadium carbide is known, for its hardness and other unique properties, as a refractory material. The synthesis of vanadium carbide is always associated with the utilization of expensive active metals, such as aluminum, calcium and magnesium, as a reducing agent to extract the vanadium metal from its corresponding oxide, followed by carbidization. The carbidization of reduced vanadium requires a complicated process and elevated temperature. Mechanical activation to synthesize vanadium carbide from its corresponding oxide and carbon source represents a promising, straightforward and less energy-intensive route. In the present study, vanadium carbide is synthesized by the carbothermic reduction of a mechanically activated mixture of V_2_O_5_ and carbon black as reducing agents without any additives. The reduction process is monitored by means of thermogravimetric analysis. The reduction products are characterized by X-ray diffraction and field emission scanning electron microscope. It is found that V_8_C_7_ with an average crystallite size of 88 nm can be synthesized from a V_2_O_5_-C mixture after milling for 15 h and further heating at 1050 °C for 1 h in an inert atmosphere.

## 1. Introduction

Transition metal carbides have been known for their importance in different fields, such as metallurgy, electronics, refractory applications, cutting tools and catalysts, because of their various unique properties, such as high hardness, high melting point, high temperature strength, high thermal conductivity and high chemical stability [1,2,3,4,5,6]. Vanadium carbide (VC), an interstitial carbide derived from the (5A) transition metal category, has favorable properties, such as a high melting point (2684 °C), a hardness of 9–9.5 Mohs, an elastic modulus of about 380 GPa, chemical stability and high electrical conductivity [7,8,9]. It has attracted much attention as a material for structures exposed to high temperatures, for surface protection and for wear-resistant parts, especially when used in a corrosive environment. Therefore, it is suitable for several applications, for example, cutting tools and abrasive and anti-wear materials [10,11]. It has been commonly used in matrix or coating materials as the reinforcement phase to enhance the wear resistance of metal materials [12]. The uniformly distributed large quantity of spherical or lumpy VC in high-speed steel enhances its ability to resist abrasive Al_2_O_3_ micro-cutting [13].

Traditionally, vanadium carbide is prepared by various methods, such as (i) the low-pressure reaction of vanadium hydride with carbon in a reducing atmosphere at 2000 °C, (ii) the vacuum reaction of elemental vanadium and carbon at a high temperature, (iii) the carbothermic reduction of vanadium trioxide or pentoxide in the presence of active metals (i.e., aluminum, magnesium and calcium) and (iv) the melting of vanadium metal in the presence of carbon. These methods are rather complicated and either energy intensive or rely on costly raw materials [3,14]. Recently, other methods have been developed to synthesize vanadium carbide powder, including (i) temperature programmed reactions [15,16], (ii) the aluminothermic reduction of vanadium oxide [17,18], and iii) the carburization of vanadium oxide with an organic reagent such as cyanamide [19]. Due to their high cost and their limited yield, these methods are still industrially inapplicable.

In the present study, an innovative route to synthesize vanadium carbide by means of the mechanical activation of a mixture of vanadium oxide and carbon is developed. The milling of a metal oxide–carbon mixture is an economical and effective method that would decrease the energy needed for oxide reduction, and could be used in forming the corresponding metal carbide in the presence of sufficient carbon [20,21,22]. Few studies have investigated the synthesis of vanadium carbide by the mechanical activation of corresponding oxides. Zhang and Li [23] investigated the synthesis of vanadium carbide by ball milling V_2_O_5_, Mg and graphite powders at room temperature. They obtained VC_x_ after milling the mixture for 36 h without external heating. The formed VC_x_ was then transformed to V_8_C_7_ after annealing at 950 °C. Hossein-Zadeh and Mirzaee [3] synthesized V_8_C_7_ through high-energy ball milling of vanadium pentoxide, calcium and carbon black mixture. After milling, the mixture was then microwave heated. They have concluded that V_8_C_7_ can be prepared after milling for 15 h and microwave heating at 800 °C for 10 min. It is worth noting that the starting mixtures in these studies include active metals such as magnesium, calcium and aluminum. Although these methods are simple, the method is still uneconomical due to the high price of active metal powders. This disadvantage opposes the main target of the mechanical activation process. In order to keep this process feasible, the utilization of such active metal powders has to be avoided.

Although the milling or mechanical activation concept is not new in producing alloys or carbides of vanadium from their corresponding oxides, the literature discussing the formation of vanadium carbide from vanadium oxide and carbon with no added active metal powder is missing. Therefore, the present paper focuses on the synthesis of vanadium carbide by mechanical activation of a V_2_O_5_-C mixture, followed by heating under controlled conditions. The structure evolution of the produced material is identified using different analytical tools. Moreover, the effects of process parameters such as the mechanical activation condition, the temperature and the time on the synthesis process and the product are also investigated.

## 2. Materials and Methods

### 2.1. Materials

The starting materials used in the present study were (i) commercially available powders of V_2_O_5_ (purity of 99% and mean particle size of 200 μm) purchased from Loba Chemie, Mumbai, India, and (ii) high-purity carbon black powder (99%) with mean particle size 6 μm, which is used as a reducing agent, purchased from Alexandria carbon black company, Alexandria, Egypt. The particle size distribution was measured using a laser diffraction analysis (BT-2001 laser particle size analyzer, Bettersize instruments, Liaoning, China). The instrument has a particle size measurement range of 0.1 to 1000 μm. Vanadium pentoxide was mixed thoroughly with excess carbon (1.5 × stoichiometry) to provide sufficient carbon to ensure the complete reduction of V_2_O_5_ to vanadium carbide according to the following reaction:

V_2_O_5_ + 7C → 2VC + 5CO (1)

### 2.2. Procedure

The V_2_O_5_-C mixture was milled for different milling periods (5–15 h) in a vertical planetary ball mill with an agate cell containing silicon nitride balls of 20 mm diameter. A ball powder ratio (BPR) of 10:1 and a rotational speed of 100 rpm were maintained throughout the whole study.

**Figure 1:**
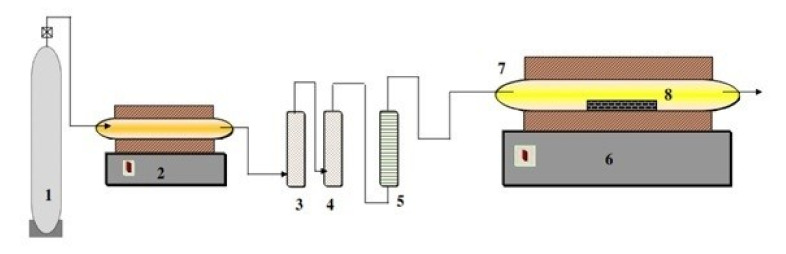
Schematic diagram for the experimental setup for vanadium carbide synthesize, 1—Argon gas cylinder; 2—Furnace containing copper turnings; 3—Pyrogallol; 4—Silica gel granules; 5—Flow meter; 6—Horizontal tube furnace (maximum temperature 1300 °C); 7—Fused alumina tube; 8—Fused alumina boat (sample holder).

The reduction experiments (synthesis of VC) were carried out in a horizontal tube furnace, a schematic representation of which is depicted in Figure 1. This consisted of an electrically heated horizontal tube furnace with an alumina tube of 100 cm long and 3 cm inner diameter. The alumina tube was inserted into the furnace and closed by rubber stoppers with gas inlet–outlet connections. The even temperature zone under this setup was measured to be ~10 cm.

Approximately 2 g of the mechanically activated powder was transferred to a clean fused alumina boat and inserted into the even temperature zone of the furnace. To maintain the oxygen potential in the reaction compartment at the minimum, the residual oxygen, H_2_O and CO_2_ in the used argon were further removed by passing the gas through heated copper turnings, pyrogallol, and silica gel, respectively. After introducing the sample, the reaction tube was flushed with purified argon to sweep off the residual air in the reaction tube. The sample was then heated at a constant heat rate (10 °C/min) to the desired temperature (1000–1150 °C) and the reduction process was allowed to continue for a definite period. A continuous flow at 100 mL/min of purified argon was maintained throughout the whole thermal cycle. At the end of each experiment, the product sample was rapidly cooled by moving to the gas cooled zone and flushing the sample with purified argon to avoid any oxidation of the reaction product. After cooling down to room temperature, the reaction product was discharged and kept in a desiccator for further characterization.

### 2.3. Characterization

Thermal analysis of the activated mixture was conducted by means of heating the mixture under an inert atmosphere at a constant heating rate of 10 °C/min, up to 1200 °C using a Bahr thermal analysis STA 504 instrument with a detection limit of ± 0.5 g. The phase compositions of the starting materials as well as the reaction products were analyzed by XRD (XRD, Bruker axs D8, Germany) with Cu kα radiation (λ = 1.5406 Å) and a secondary monochromator in the 2θ range from 10° to 80°. Phase identification for the examined samples was carried out using JCPDS (Joint Committee on Powder Diffraction Standards) cards while their morphology was investigated by field emission scanning electron microscope (FESEM, Quanta, FEG 250) in the secondary electron (SE) mode and with an accelerating voltage of 20 kV.

## 3. Results and Discussion

### 3.1. Thermogravimetric Analysis

**Figure 2:**
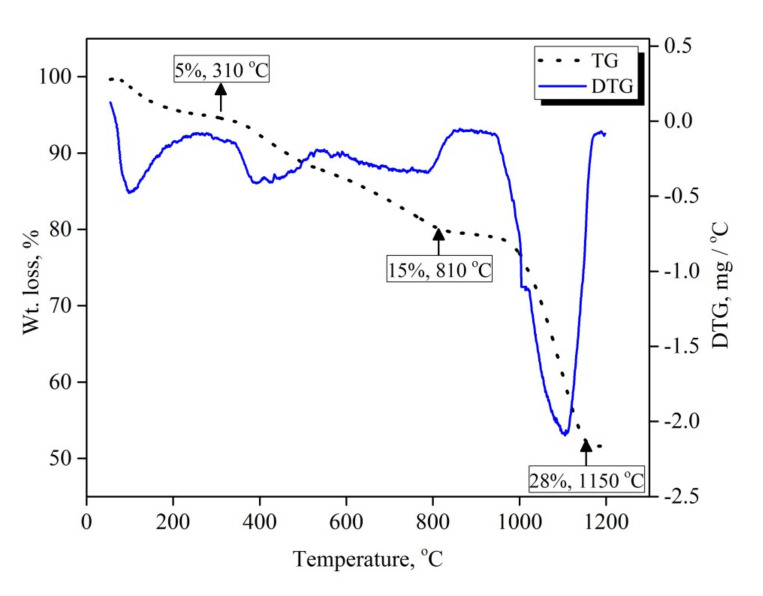
TGA-DTG curves of 15 h mechanically activated V_2_O_5_-carbon black mixture.

Figure 2 shows the mass loss (TG) and the first derivative of mass loss (DTG) as a function of the temperature of the mechanically activated V_2_O_5_-carbon mixture. The sample was heated from room temperature up to 1200 °C at a heating rate 10 °C min^−1^ under an argon atmosphere. A net mass loss of 48 wt. % was observed, the mass loss in this case being a result of de-moisturization and the release of reducible oxygen and carbon. TG and DTG curves show three distinguishable different reaction steps, indicated by the slope change over the course of the reaction. The first step was a gradual mass loss with a very slow rate, which started from 100 °C and proceeded up to 310 °C with a mass loss of 5 wt. %. This initial slow mass loss rate can be attributed to the desorption of adsorbed moisture during the mechanical activation process. The second mass loss step started from 310 °C and continued to 810 °C, with a mass loss value of 15 wt. % which can be related to the slow solid–solid reaction between carbon particles and V_2_O_5_ to produce VO_2_. This mass loss value matches nicely with the theoretical weight loss when V_2_O_5_ is reduced to VO_2_ by carbon (14.4 wt. %). The mass loss appeared to level-off between 810 °C and 950 °C, and then started to increase again above 950 °C. This behavior could be attributed to the effect of the formed VO_2_, which produced a thick layer hindering the diffusion of carbon monoxide gas away from the reaction site and consequently retarded the reaction progress. Increasing the temperature resulted in a significantly increased mass loss rate until it approached 28 wt. % mass loss at 1150 °C, which is due to the continuation of the reduction of VO_2_ to VC.

### 3.2. Effect of Reduction Temperature

**Figure 3:**
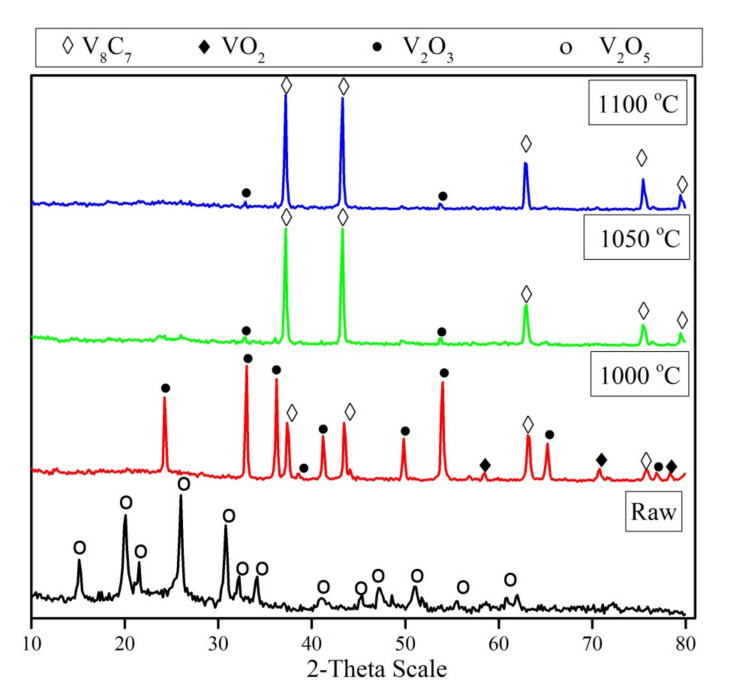
XRD patterns of un-milled, and reduction products of 15 h milled, V_2_O_5_-C mixture at temperature range 1000–1100 °C for 1 h.

Figure 3 displays the XRD patterns of the mechanically activated V_2_O_5_-C mixture and its carbidization products at different temperatures. It is obvious that the temperature has a considerable effect on the formation of vanadium carbide. The XRD patterns of the mechanically activated V_2_O_5_-C mixture prior to heat treatment show only V_2_O_5_ peaks, while no peaks corresponding to carbon could be detected. The absence of carbon peaks can be attributed to the amorphization of carbon due to mechanical activation. Moreover, the only observed difference between the starting vanadium pentoxide patterns and the mechanically activated V_2_O_5_ in the mixture was the broadening of peaks, which can be explained by the reduction in the mean particle size of the mixture which is, in turn, a result of milling during the extended duration. Furthermore, the reduction in mechanically activated mixture at 1000 °C gave rise to the formation of a V_2_O_3_ phase, with small amounts of vanadium carbide, and traces of a VO_2_ phase. By raising the temperature up to 1050 °C, the intensity of V_2_O_3_ decreases sharply and, in parallel, high intensity peaks of vanadium carbide were observed without the presence of any intermediates of other vanadium oxides. So, the XRD patterns of partially reacted mixtures suggest that during the reduction process, the powders undergo the following phase transformations: (i) V_2_O_5_ transforms to V_2_O_3_ and a minor amount of VO_2_, and (ii) the vanadium lower oxide transform to the corresponding carbide (V_8_C_7_). No diffraction line of VO phase is observed in the diffraction pattern, which confirms that the phase transformation does not obtain from V_2_O_3_ to VO. A further increase in reduction temperature to 1100 °C has no detectable effect on the formed phase, unless one increases the intensity of the formed vanadium carbide phase due to crystal growth. The average crystallite size of the formed vanadium carbide increases from 60 nm at 1000 °C to 88 nm at 1100 °C.

### 3.3. Effect of Reduction Time

**Figure 4:**
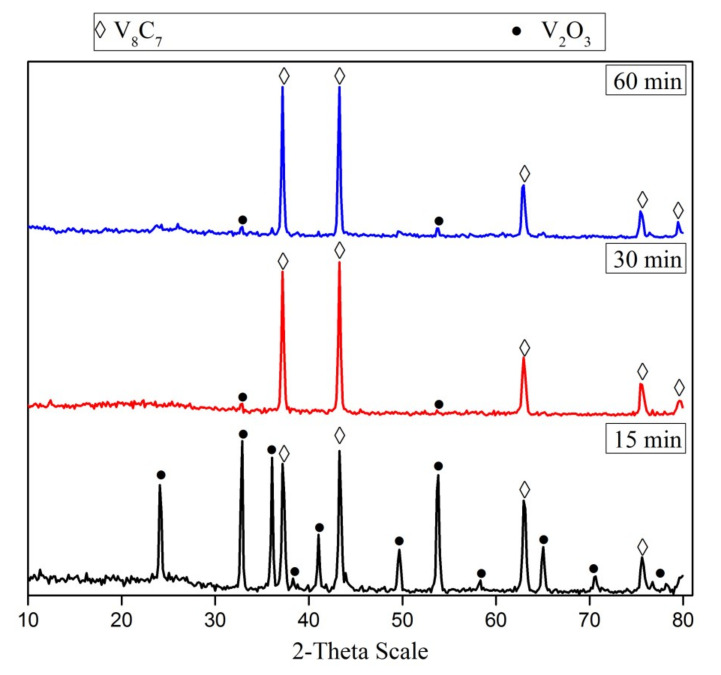
XRD patterns of 15 h mechanically activated V_2_O_5_-C mixture reduction products at 1050 °C for different reduction periods.

The effect of the reduction time of the V_2_O_5_-C mixture (milled for 15 h) was investigated at 1050 °C. Figure 4 presents the XRD patterns of phases formed during the carbidization process of the V_2_O_5_-C mixture at 1050 °C. It can be noticed that the carbide formation increases along with increasing the reduction time from 15 min up to 30 min. A further increase in reduction time had no significant effect on the formation of vanadium carbide, unless one increases the crystallite size from 80 nm at 15 min to 88 nm at 60 min.

### 3.4. Effect of Mechanical Activation

**Figure 5:**
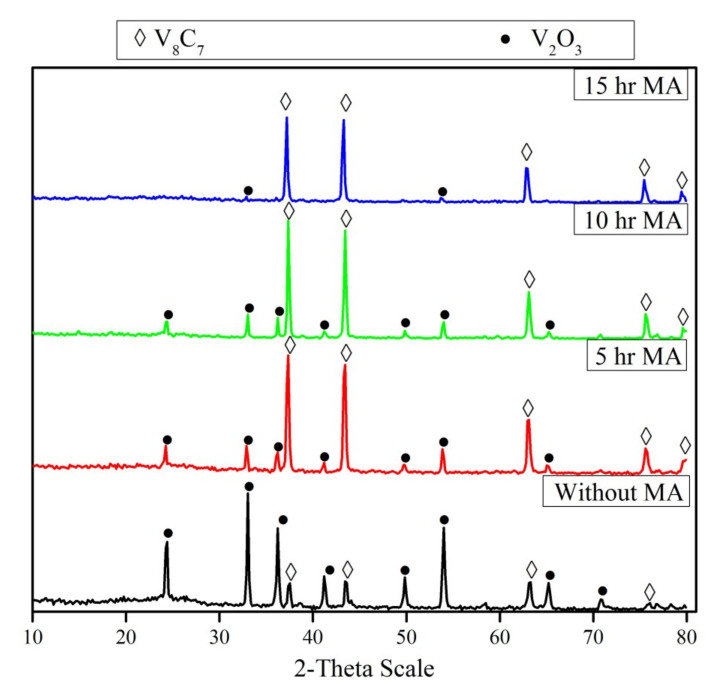
XRD patterns of reduction products of un-milled and milled V_2_O_5_-C mixture at different periods (5–15 h) after reduction at 1050 °C for 1 hr.

The un-milled and milled powder mixtures during different periods were heated up to 1050 °C for 1 h in an inert atmosphere. The XRD patterns of the reaction products are shown in Figure 5. The XRD pattern of the un-milled mixture after the reaction shows the presence of strong V_2_O_3_ peaks and traces of vanadium carbide (V_8_C_7_). On the other hand, it is obvious that mechanical activation has a significant effect on the reduction and carbidization of V_2_O_5_. Comparing the XRD patterns of milled mixtures during different periods (5–15 hr), it can be observed that the intensity of V_8_C_7_ increases along with increasing the milling periods. The intensity of the V_2_O_3_ peaks decreases with increased milling time until it disappears in the reaction products of the mixture milled for 15 h. In all cases, no lower vanadium oxides (rather than V_2_O_3_) were detected, irrespective of milling duration. The average crystallite size of vanadium carbide prepared from a 5 h milled mixture (70 nm) is lower than that of the 15 h milled mixture (88 nm).

### 3.5. Microstructure Characterization

**Figure 6:**
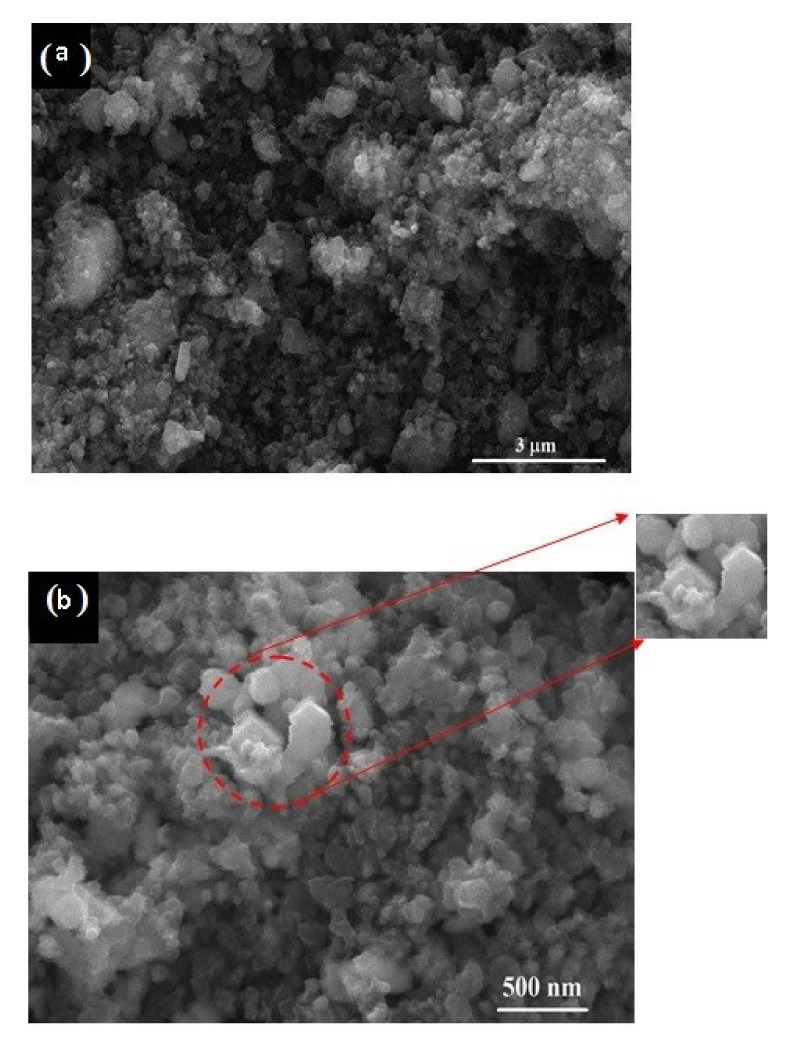
SEM micrograph of 15 h milled V_2_O_5_-C mixture (**a**) and its reduction product at 1050 °C for 1 h (**b**).

Figure 6 shows the FESEM micrographs of the 15 h mechanically activated mixture (Figure 6a) at 30,000 × magnification, with the scale bar representing 3 μm, and its reduction product obtained at 1050 °C for 1 h (Figure 6b) at 120,000 × magnification, with the scale bar representing 500 nm. Before the carbidization process (after milling for 15 h), the SEM micrograph (Figure 6a) shows the well-mixed fine particles of V_2_O_5_ and carbon black where the average particle size of the V_2_O_5_-C clusters was below 3 μm, which indicates high surface area, and thus increases the interfacial surface area between the reactants while shortening the diffusion paths. These enhancements, in addition to the created stresses due to friction and collision during milling, lead to lowering the carbidization temperature and improving the reaction kinetics. The morphology of the sample synthesized at 1050 °C for 1 h (Figure 6b) shows particles of a nanometric size (less than 500 nm), and agglomerations exist in cubic or spheroid shapes and consist of multiple particles.

### 3.6. Thermodynamic Calculation

**Figure 7:**
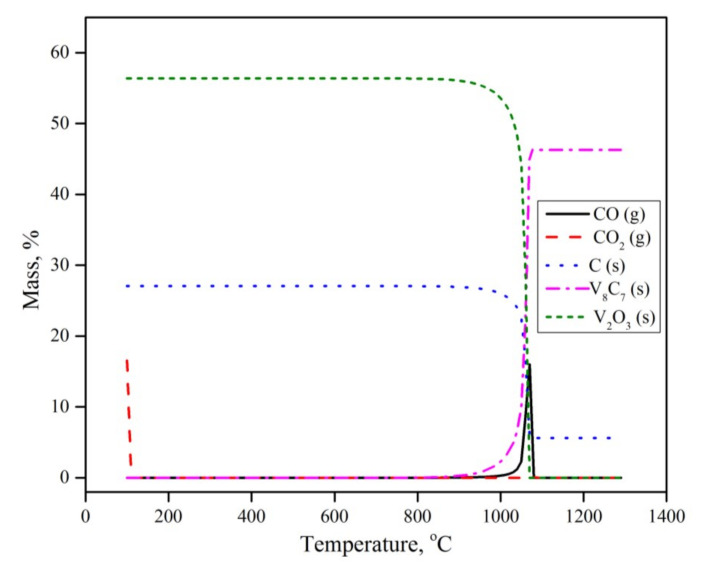
Thermodynamic calculations using FactSage.

To further understand the reaction mechanism and explain the experimental results, a set of calculations was conducted using the Equilibria module in the thermodynamic software FactSage 7.3™, developed by GTT (Herzogenrath, Germany) and CRCT (Quebec, QC, CA) [24]. In the calculations, an open system configuration was considered. In the open configuration, the product gas is continuously taken away from the system and is not considered in the following calculations. The data bases used were FT oxide, FT misc and SGPS (oxide, miscellaneous, pure substances databases respectively). The obtained calculations are given in Figure 7.

The equilibrium calculations revealed that in the presence of carbon and argon as a carrier gas, V_2_O_5_ is not thermodynamically stable, and will dissociate into V_2_O_3_ and O_2_ in the ambient temperature and pressure. O_2_ then reacts with solid C and forms CO_2_, according to Equation (2)

V_2_O_5_ (s) + C (s) → V_2_O_3_ (s) + CO_2_ (g) (2)

This observation does not agree with either the thermogravimetric or XRD results. Such a contradiction indicates that the early transformation of vanadium pentoxide is limited by reaction kinetics rather than equilibrium. The thermogravimetric results showed that V_2_O_5_ reduction to V_2_O_3_ occurs under the present conditions, in the temperature range of 300 to 800 °C. According to the present equilibrium calculations, CO_2_ will leave the system without any further reaction. V_2_O_3_ is the only thermodynamically stable vanadium oxide phase throughout the experiment, until temperatures exceed 900 °C. Above 900 °C, V_2_O_3_ transforms into V_8_C_7_ with no intermediates of other oxide or metallic phases. This observation agrees nicely with the experimental results.

It is worth mentioning that some minor XRD peaks corresponding to VO_2_ were observed, while the equilibrium calculations showed no sign of its formation. This can be explained by the fact that under a specific condition of oxygen partial pressure and temperature, the solid solutions of VO_2_ and V_2_O_3_ might coexist. However, this phase is not thermodynamically stable under the present experimental conditions.

## 4. Conclusions

In the present study, we attempted to synthesize vanadium carbide from its corresponding oxide and carbon as the sole reducing and carbidizing agent, respectively. The following points can be concluded:

1. Under the conventional conditions of the carbothermic reduction of vanadium oxide, a very small portion of the oxide can be converted to carbide, resulting in a very poor yield.
2. The mechanical activation-assisted carbothermic reduction of vanadium oxide, on the other hand, gives promising results, with a very high yield of about 95%.
3. The effects of reaction parameters like temperature, reduction duration and mechanical activation duration on the formation of vanadium carbide and the final product’s properties were investigated, and the optimum conditions were identified.
4. V_8_C_7_ nanoparticles with a crystallite size of 88 nm were prepared from a mechanically activated V_2_O_5_-C mixture after milling for 15 h and further heating at 1050 °C for 1 h.
5. The experimental results at and above 950 °C were successfully explained by equilibrium calculations. At lower temperatures, the reactions were kinetically limited.
